# Alignment-invariant signal reality reconstruction in hyperspectral imaging using a deep convolutional neural network architecture

**DOI:** 10.1038/s41598-022-22264-3

**Published:** 2022-10-19

**Authors:** S. Shayan Mousavi M., Alexandre Pofelski, Hassan Teimoori, Gianluigi A. Botton

**Affiliations:** 1 McMaster University, Materials Science and Engineering, Hamilton, L8S 4L8 Canada; 2 Brookhaven National Laboratory, Upton, NY 11973 USA; 3 McMaster University, Walter G. Booth School of Engineering Practice and Technology, Hamilton, L8S 4M1 Canada; 4 Canadian Light Source, Saskatoon, S7N 2V3 Canada

**Keywords:** Applied physics, Electronics, photonics and device physics, Information theory and computation, Optical physics, Plasma physics, Quantum physics, Techniques and instrumentation, Electrical and electronic engineering, Energy science and technology, Engineering, Materials science, Nanoscience and technology, Optics and photonics, Physics

## Abstract

The energy resolution in hyperspectral imaging techniques has always been an important matter in data interpretation. In many cases, spectral information is distorted by elements such as instruments’ broad optical transfer function, and electronic high frequency noises. In the past decades, advances in artificial intelligence methods have provided robust tools to better study sophisticated system artifacts in spectral data and take steps towards removing these artifacts from the experimentally obtained data. This study evaluates the capability of a recently developed deep convolutional neural network script, EELSpecNet, in restoring the reality of a spectral data. The particular strength of the deep neural networks is to remove multiple instrumental artifacts such as random energy jitters of the source, signal convolution by the optical transfer function and high frequency noise at once using a single training data set. Here, EELSpecNet performance in reducing noise, and restoring the original reality of the spectra is evaluated for near zero-loss electron energy loss spectroscopy signals in Scanning Transmission Electron Microscopy. EELSpecNet demonstrates to be more efficient and more robust than the currently widely used Bayesian statistical method, even in harsh conditions (e.g. high signal broadening, intense high frequency noise).

## Introduction

In a general sense, hyperpsectral imaging can be referred to all techniques exploring spectral properties of a specimen locally. These techniques may utilize different excitation sources such as photons or electrons to interact with a specimen of choice. Hyperspectral imaging techniques are widely used to study wide range of properties such as vibrational, optical, and chemical properties^1–6^. Although these techniques are continuously evolving, the acquired spectra suffer from different signal distortion phenomena at different levels depending on the particular technique. The signal distortion in these spectroscopy techniques are usually a combination of high frequency noise (HF) and signal convolution due to the optical transfer function (OTF) of the instrument used to collect the data^7–10^. In this regard, spectral deconvolution is necessary for extracting fine spectral features and also quantitative analysis of the data. Among different deconvolution techniques implementation of partial differential equation-based and filter-based techniques has been ubiquitous^11–21^. However, both these deconvolution paths have some drawbacks. In the case of deconvolution through partial differential equation techniques, the models usually require detailed information about the signal and physical phenomena that, in many cases, are not available. In the realm of deconvolution through filter based techniques, such as Fourier-based methods and iterative Bayesian methods, high frequency noise-related artifacts are usually increased. The removal of HF noise and other signal artifacts become extremely important when the signal to noise ratio in the target signal is already low and quantitative analyses on the shape and bandwidth of peaks or other signal modulations are required.

Near zero-loss peak (near-ZLP) electron energy loss spectroscopy (EELS) is an example of a hyperspectral electron microscopy technique which, in addition to spectral convolution, greatly suffers from high frequency noise, and strong background signal generated by the tail of a major peak called zero-loss peak (Fig. S1). These artifacts bury many peaks containing significant information about vibronic, phononic, and surface plasmonic activities in different media and make accurate quantitative analyses almost impossible^21–24^. Indeed, the EELS technique offers both unrivaled high spatial resolution (nanometers and sub-nanometers range) and high energy resolution (down to a few meV) which are crucial for design and studying fields including optoelectronics, photonics, biosensing, imaging, and plasmon-mediated therapies^22,25–31^. Thus, any improvement in the EELS technique has a direct impact on fields such as nanoscale electronic and photonic structures. However, the convolution problem in electron microscopy-based techniques can be more complicated than a simple OTF broadening. In the case of EELS, the electron beam (i.e. the excitation source) experiences some energy instabilities which results in high and low frequency energy shifts of the spectra which will be averaged during the detector’s exposure time. As the detector’s recording process is cumulative, the output spectrum is a sum of all energy-shifted spectra recorded during the exposure time, introducing another broadening mechanism to the EELS signals^32^. Due to the random nature of these energy jitters, and their dependence to experimental condition, statistical methods (filter-bases methods) and partial differential equation-based methods cannot accurately conduct spectral deconvolution tasks.

In recent years, machine learning (ML) algorithms have tackled problems with computational complexities beyond capacity of conventional techniques.The dimensionality reduction techniques such as principal component analysis (PCA) are an example of ML solutions used for extracting main features from distorted signals^33–35^. Although ML methods, especially deep learning (DL), have been extensively used for image and signal deconvolution or feature detection and classification^36–43^, their capability in dealing with spectral features (broadened, low dose features) that may have scientific significance in an extremely distorted signal is less investigated particularly for near zero-loss EELS signals and in terms of validating physical reality of the signal. In this regard, publications towards low-loss EELS signal processing are mainly limited to study either denoising the signal or improving the background signal^44–47^.

In this work, it is proposed to use the complete power of DL to reconstruct the physical origin of the EELS signal by focusing on removing all artifacts at once. Being able to effectively retrieve fine meaningful features from a distorted signal opens doors to areas such as low-dose spectroscopy, ultra-fast microscopy, and single pixel-based analysis. Moreover, a robust spectral restoration method that can retrieve distorted signal in different conditions is a solution for alignment-invariant microscopy, mitigating human errors and instrumental fluctuations and limitations. For this purpose, a U-shaped fully convolutional deep neural network (U-CNN) with skip connections (concatenations between different layers of network), scripted in Python, named EELSpecNet^48,49^, is used to reconstruct the original reality of the EELS hyperspectral information from what is recorded on the instrument’s detector (e.g. a charge-coupled device, CCD). Application of different U-shaped networks are mainly investigated for image segmentation and classification tasks and, to a smaller extent, signal denoising^40–43^. Here, a further step is taken by evaluating EELSpecNet’s U-shape network for signal deconvolution and reality reconstruction. In this scenario, features generalization is used for producing training sets for preventing the DL neural network to converge to a specific simulated model, leaving the opportunity to resolve other phenomena existing in spectral data. Feature generalization is a generative approach based on a random selection of parameters representing different components of the signal and is further discussed in the training strategies and pipelines section.

The following sections introduce EELSpecNet’s deep U-CNN architecture, describe training strategies, training performance, and evaluate different aspects of signal restoration power of the network including noise reduction, background removal and signal fidelity. To appreciate the benefits from EELSpecNet, all the evaluations are compared with a widely used low-loss EELS Bayesian deconvolution technique, namely the Richardson-Lucy method^19–21^.

## Results

**Figure 1:**
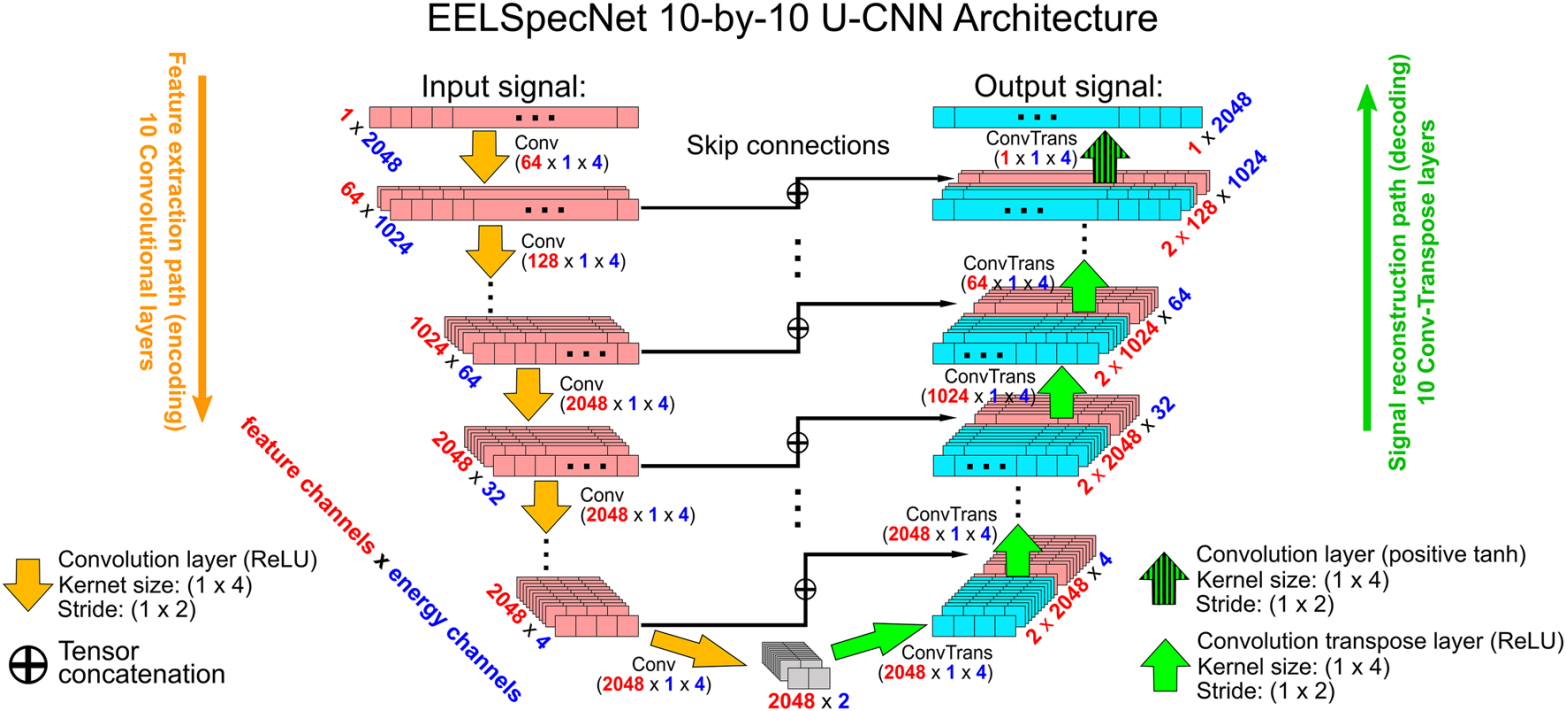
EELSpecNet 10-by-10 U-shaped fully convolutional neural network architecture with 10 layers for feature extraction (encoding) and 10 layers for signal reconstruction (decoding). As is demonstrated, input and output are assumed to have 2048 energy channels (pixels) and encoding and decoding layers are connected by skip connections.

In this section, different aspects of the EELSpecNet U-CNN signal restoration performance in restoring near zero-loss EELS (NZ-EELS) signal are presented. Figure 1 presents the U-shaped convolutional neural network with 10 encoding and 10 decoding layers with total of 218,094,209 trainable parameters. Each of the encoding layers is connected (concatenated) to its corresponding decoding layer to improve the learnability of the network^50,51^. The number of encoding and decoding layers in the architecture can be different, 10 layers are selected to establish a balance between computation time and model accuracy; Figure S2 demonstrates an examples of trained networks with different number of layers. This neural network architecture can be generically applied to any relevant case and is not exclusive to EELS signal. EELSpecNet network is heavily inspired by U-net^39,42,43^. Instead of using the U-shaped CNN for classification and segmentation, however, here, the network is adjusted for the spectral deconvolution and signal reconstruction tasks; Figure 1.The implementation and a brief investigation over the performance of the different depths of the EELSpecNet neural network can be found in the devoted GitHub repository to this script^49^. In the following, training strategies and performance of the network presented in Fig. 1 (10-by-10 network) are discussed.

### Training strategies and pipelines

Unlike in classification and segmentation tasks for which the solutions (correct labels) are usually known, the ground truth in most deconvolution tasks is not available experimentally. In some scenarios, parallel experiments may provide data with improved resolution but pixel by pixel matching of the data is almost impossible. Moreover, it could be tempting to use a representative experimental data as an image of the ground truth (such as an EELS spectra in vacuum), however, such approaches limit the deconvolution process to the instrumental resolution. Experimentally, there is indeed no access to the original signal not distorted by the OTF of the instrument. There are also methods using simulated data as the ground truth which, other than dictating a theoretical model bias to the neural network, may make the execution extensively time consuming. For instance, the surface plasmon activities are highly related to the specimen’s geometry and the materials used. In the best case scenario, simulations can only be done for specific shapes which may not fully follow the real particle’s spatial details and artifacts.

In this study, feature generalization is one solution to mitigate biases from a subjective theoretical model. In this regard, the ground truth and its instrument-related distortions are simulated based on random events to generate data for the training of the deep neural network. The boundaries of the signal distortion mechanisms are defined in a way to contain representative instrument-related phenomena and also cover a wider range of general features expected in the signal. In other words, by loosely defining the features in different components of a signal, and increasing their degree of freedom, part of the training set is intentionally undergoing stronger or weaker distortions than the target signal (experimental); we define this approach as generalized-learning.

Using this generalized-learning drastically simplifies and accelerates the test set preparation when no specification about the ground truth is available, or only some general information about a signal is known but exact behavior is still unknown. In addition, such generalized-learning can reduce the training set biases by introducing the option to discover features not originally expected in the signal.

**Figure 2:**
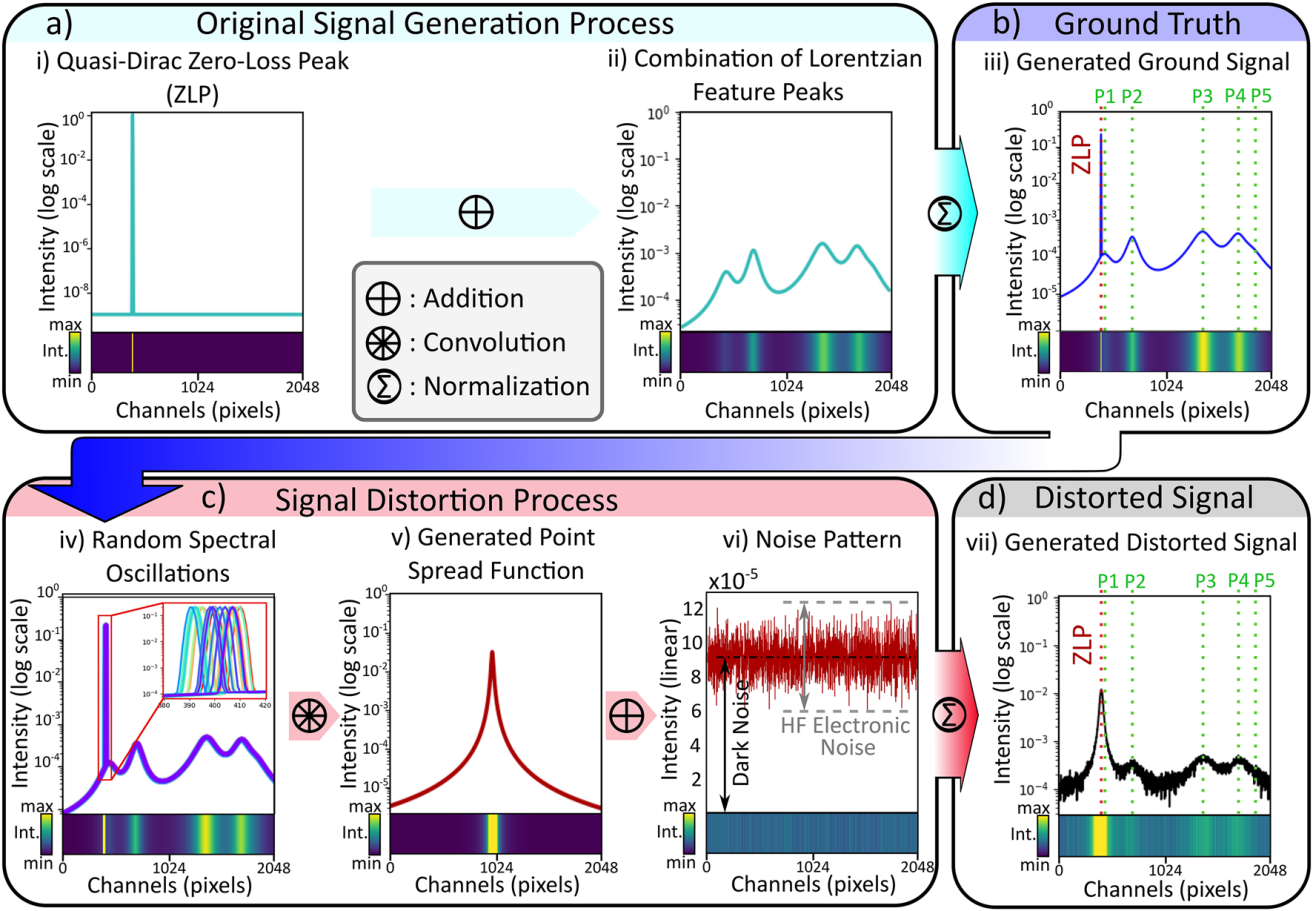
A schematic illustration of data generation pipelines for NZ-EELS generalized-learning. Each signal is also shown using energy resolved heat maps below them for better visualization of the data. (**a**) A demonstration of ground truth generation using a quasi-Dirac function and a combination of Lorentzian peaks. (**b**) The ground truth signal, normalized by the integral of the signal. (**c**) Signal distortion steps including energy jitters of the beam, optical transfer function broadening, and high frequency noise. (**d**) The distorted signal, normalized by integral of the signal.

In order to implement generalized-learning, this study focuses on NZ-EELS signals and artifacts that are introduced in each spectrum in the electron microscope experiment. In this regard, the ground truth of a NZ-EELS signal can be divided into 2 components; the zero-loss peak, and peaks related to low-energy signals that have around 1000 times less intensity than the zero-loss peak (e.g. signals from phononic, and plasmonic activities). The sources of signal distortions in EELS spectra include: convolution by the instrument’s optical transfer function (OTF, also known as point spread function, PSF), spectral energy wobbles due to electronic or mechanical instabilities during the exposure time, and high frequency noise from electronics of the instrument^32,52–54^. The EELSpecNet data generation pipelines construct signals with similar components as in an original EELS signal and adds more modulations and randomized features (both more and less intense features than the real data); see Fig. 2.

In order to train the EELSpecNet U-CNN, the ultimate desirable ZLP in the ground truth signal is a Dirac delta function. This assumption is equivalent of having a perfect electron beam with no energy deviations. However, to prevent computational difficulties, the beam is defined to be a Gaussian with 3 energy channel (a quasi-Dirac function); see Fig. 2a. The feature peaks, are assumed to be harmonic oscillatory phenomena such as surface plasmonic and phononic activities taking place at low energies. Due to the harmonic properties of these peaks they are expected to show a Lorentzian distribution in nature^24,55^; see Fig. 2a,b. To generate the distorted signal, as is shown in Fig. 2c,d, the ground signal is randomly oscillated, convoluted with a generated point spread function, and eventually sprinkled with a high frequency noise signal with a non-zero mean (known as dark noise) and different amplitudes generated by the electronics of the system.

In this work EELSpecNet is trained for a data-set containing 6000 spectra (5000 spectra for training and 1000 spectra for validation); see Table S1. Each spectrum is generated as is shown in Fig. 2. The best training outcome (final fitting and convergence during the training process) is obtained by using log loss as the loss function and Adam optimizer^56–58^. The training is also monitored by mean squared error and mean absolute percentage error as these errors are more commonly used and are easier to understand. Figure S3 demonstrates the loss and errors measured during the training and validation process. It is worth noting that such results were obtained by establishing a balance between the hyperparameters and the generalized learning approach that enabled reducing the size of the training data set.

### Performance evaluation

To evaluate the performance of EELSpecNet, the established Richardson-Lucy (RL) Bayesian iterative deconvolution method for deconvolving NZ-EELS spectra is used as a reference^19–21^. In this regard, signal reconstruction (deconvolution) quality is evaluated using different measures. First, the deconvolution result is visually inspected; then, the noise variance in the signal is calculated before and after deconvolution. Eventually, the quality of the ZLP tail removal, and fidelity of the reconstructed signal to the original reality are investigated.

**Figure 3:**
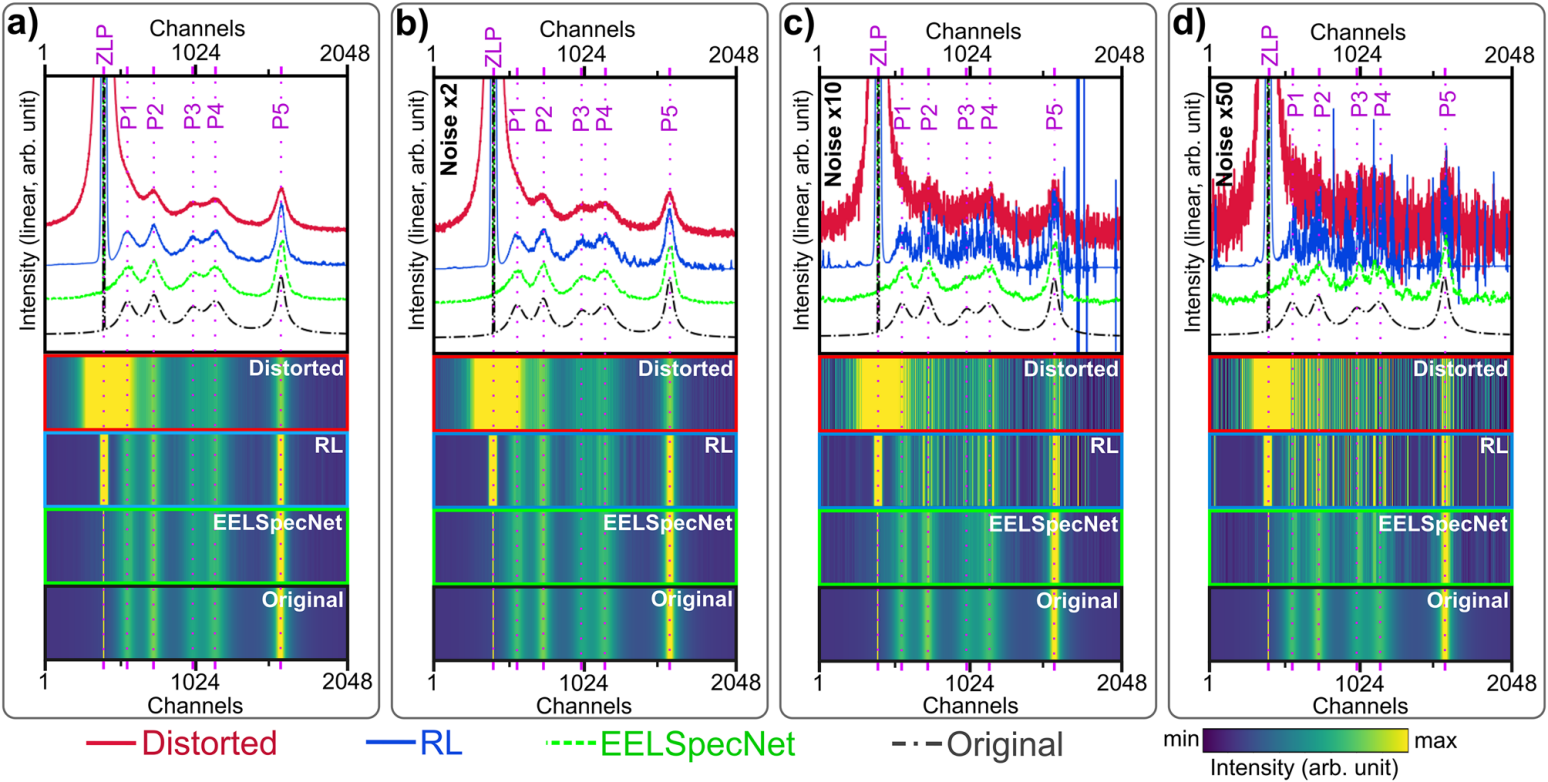
An example of NZ-EELS signals of different noise levels with (**a**) low (the noise amplitude of $5\times 10^{-6}$), (**b**) medium-low (the noise amplitude of $1\times 10^{-5}$), (**c**) medium-high (the noise amplitude of $5\times 10^{-5}$), and (**d**) high (the noise amplitude of $1\times 10^{-4}$) HF noises artifact, deconvolved using 50 iterations of RL and EELSpecNet deep U-CNN. The spectra are also shown using energy resolved colormaps for better visualization. EELSpecNet performance is stable even at highly deteriorated signal while RL-retrieved signal suffers from high frequency artifacts.

For visual inspection, generated convoluted signals with different levels of high frequency noise with a known ground truth, are deconvolved using EELSpecNet and 50 iterations of the RL algorithm. The number of 50 iterations is suggested based on Bellido’s work as a range that effectively retrieves the original signal, while not introducing excessive artifacts to the signal^21^. Figure 3 demonstrates an example of a spectrum that is convoluted with a broad optical transfer function with a full width at half maximum (FWHM) of 32 channels. For instance, if each energy channel has an energy width (dispersion in more specific terminology) of 5 meV, the FWHM of the spectrum with 32 channels becomes 160 meV which is drastically broader (worse) than what is being used for NZ-EELS purposes which is typically below 80 meV. For better visualization, spectra in Fig. 3 are shifted, and spread uniformly along the vertical axes. These spectra are normalized by the integral of all channels’ intensity.

As is shown in Fig. 3, four scenarios of noise modulations are applied to the signal which represent different experimental conditions:

- Low level HF noise with intensity amplitude of $5\times 10^{-6}$, representing a noise level below common experimental conditions (e.g. filtered data); see Fig. 3a.
- Medium-low level HF noise of amplitude $1\times 10^{-5}$, representing a noise level similar to a typical EELS spectroscopy signal noise level (not high energy resolution application); see Fig. 3b.
- Medium-high level HF noise with amplitude of $5\times 10^{-5}$, which stands for high energy resolution operations (Monochromated, low-dose spectroscopy); see Fig. 3c.
- High level HF noise of amplitude $5\times 10^{-4}$, that show a condition beyond typical high-resolution application; see Fig. 3d.

Following data processing with EELSpecNet, the main visible difference between deep learning (EELSpecNet) approach and iterative method (RL) in Fig. 3 is the intrinsic higher reliability of deep learning method in effectively restoring the signal’s ground truth, even in a spectrum with intense noise. The high restoration power in presence of noise is essential in high energy resolution imaging, especially for beam sensitive material characterization, single spectrum feature analysis to improve the locality of the captured data (spatial resolution), and conditions where enhanced resolution is a result of having low beam current, short exposure time, or using monochromated systems^32,52,59–64^.

**Figure 4:**
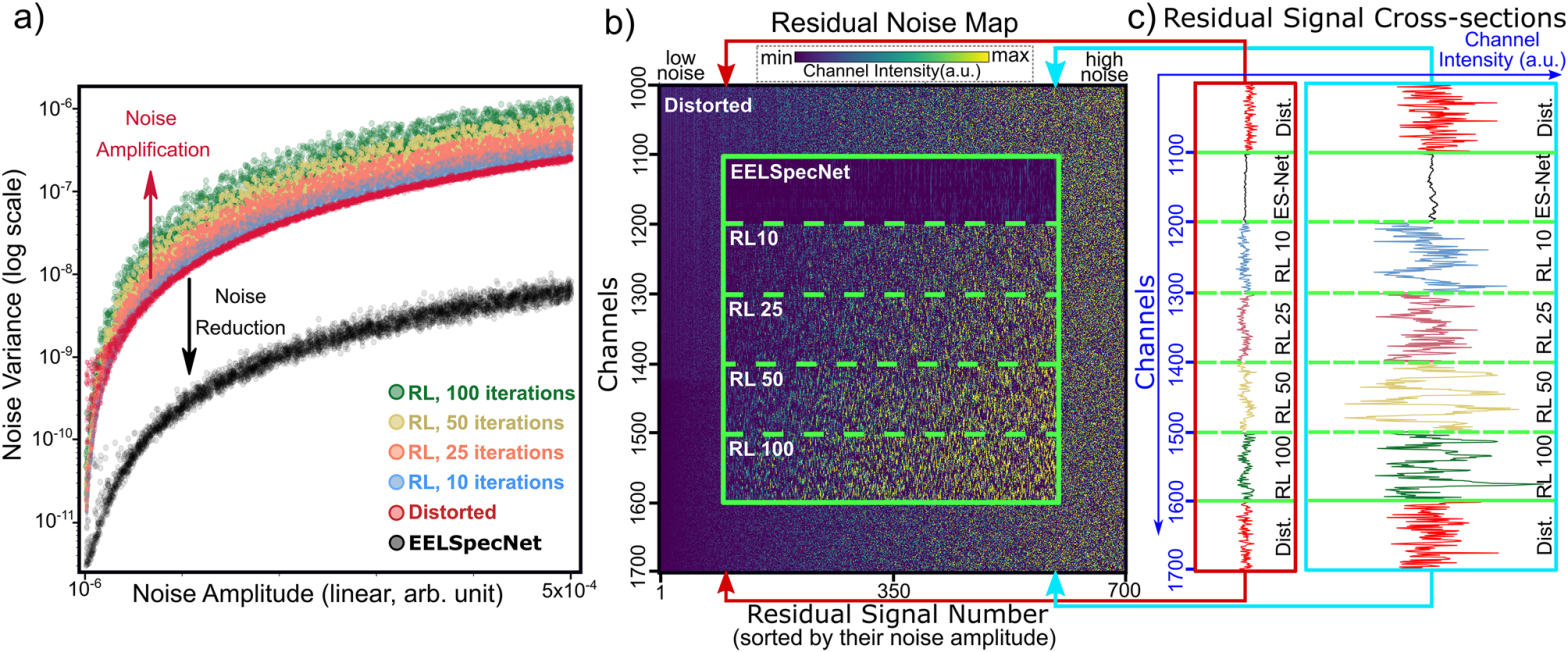
(**a**) Measured noise variance in each spectrum in a set of 4000 generated spectra before deconvolution (distorted signal) and after deconvolution using EELSpecNet and different iterations of the RL algorithm. (**b**) The residual noise map demonstrates the feature-less channels (no peaks) of the first 700 distorted spectra from the 4000 spectra generated for noise evaluation. Each spectrum is put on vertical axes and they are sorted by their noise amplitude in the distorted data. The green box represents snapshots of channels deconvolved using different methods. (**c**) slices of a residual noise map at low and high noise amplitudes are demonstrated.

For a quantitative evaluation of EESpecNet performance, the noise variance in 4000 spectra with HF noise amplitude from $1\times 10^{-6}$ to $5\times 10^{-4}$ is measured before and after deconvolution. These spectra only contain the zero-loss peak and do not have any other peaks. The noise variance is measured at channels further from ZLP. As is represented in Fig. 4, the noise variance is measured for deconvolved spectra using EELSpecNet and different iteration of the RL algorithm.

The plot in Fig. 4a shows how the noise variance changes with respect to the amplitude of HF noise in the distorted signal. Based on the results, EELSpecNet reduces the noise variance by a factor of 40, while RL increases the noise variance at worst by a factor 4. The higher the RL iterations, the higher is the noise variance in the signal. The stability of EELSpecNet performance in noise reduction is also considerable even at extremely high HF noise amplitudes; this stability is more visible in linear scale; (Fig. S4).

The comparison between the deep learning solution and commonly used RL method is also demonstrated using the residual noise color map (Fig. 4b). Each vertical column in the residual noise map in Fig. 4b displays the residual high frequency noise signal after subtracting the original reality from the distorted signal; more descriptions can be found in Fig. S4. These residual noise signals (vertical columns of the heat map, Fig. 4b) are sorted based on the amplitude of the HF noise in the distorted signal. Figure 4b, for the ease of demonstration, only displays the first 700 signals from the 4000 generated signals. Figure S4, represents a similar map for all 4000 generated signals. The inset green boxes in Fig. 4b, illustrate the equivalent residual noise on the same channels of the original residual map after applying different restoration procedures (EELSpecNet and different iterations of RL). Figure 4c shows two slices from residual noise map at a low- and a high-noise column as an example. Based on this noise evolution analysis (Fig. 4) while the currently used Bayesian deconvolution method is incapable of reducing HF noise, the deep learning solution effectively suppress the HF noise in signals with different level of degradation.

The most prominent component in a NZ-EELS signal is Zero-loss peak (ZLP). The effectiveness in the removal of the tails of the ZLP in a deconvolution process is extremely important as many low energy signals such as phononic, plasmonic, and single electron transitions take place at an energy range covered by this tail. In this study, the efficiency of ZLP tail removal is evaluated by probing the full-width at half maximum (FWHM) and full-width at tenth maximum (FWTM) of the ZLP before and after signal reconstruction. Two indices used to evaluate the effectiveness of this process are the recovery rate, and the relative error (Equation S1-4). The recovery rate, represents the rate of ZLP’s FWHM or FWTM retrieval in the restored signal in comparison to the distorted signal (Eqs. S1–S2). The relative error measures the relative error between the FWHM and FWTM of the ZLP in the restored signal compared with the original signal (Eqs. S3 and S4).

**Figure 5:**
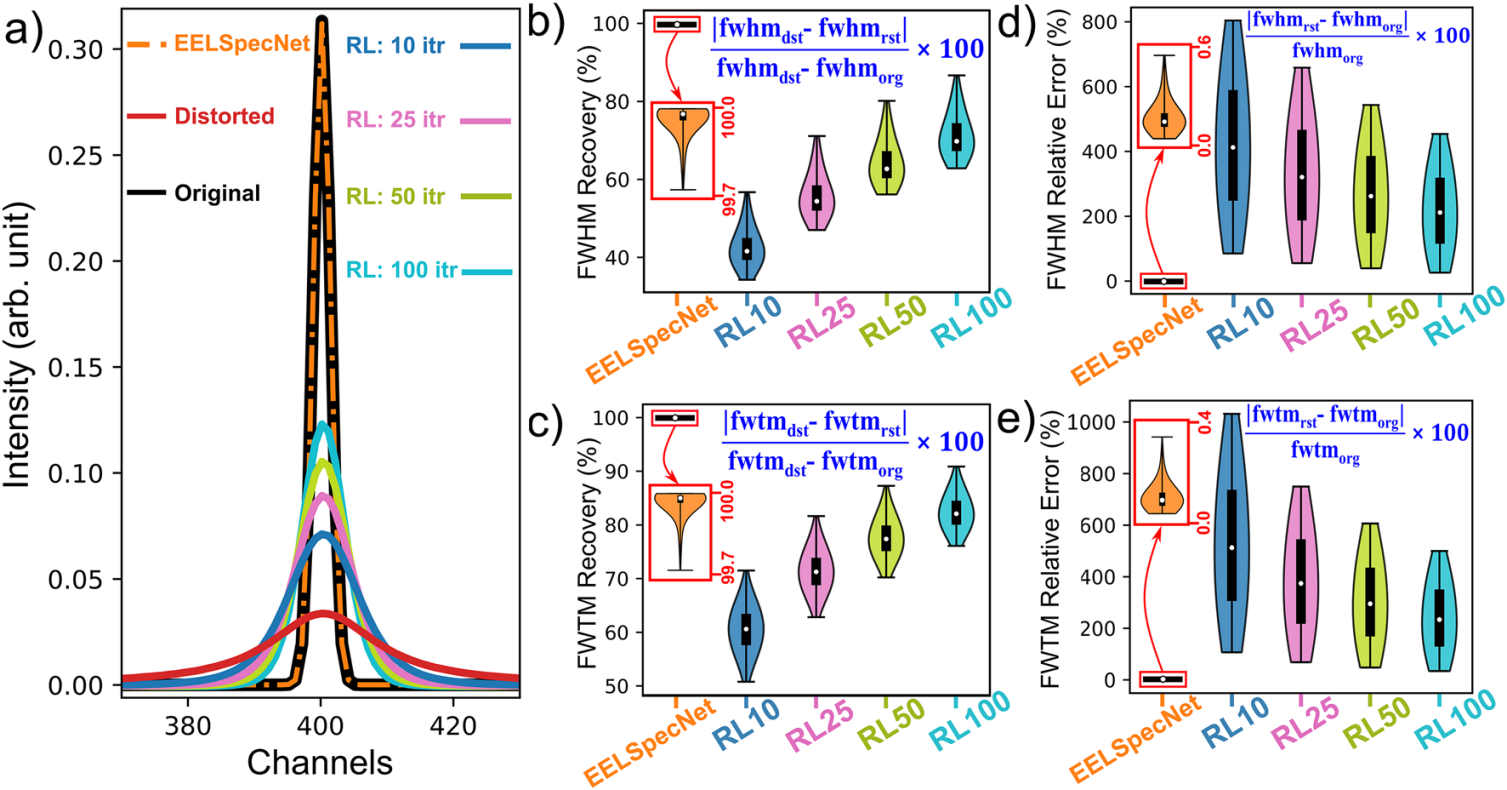
(**a**) An example of a ZLP restoration using EELSpecNet and RL (10, 25, 50, and 100 iterations). Violin plots demonstrating: (**b**) ZLP FWHM and (**c**) ZLP FWTM recovery rate, and (**d**) ZLP FWHM and (**e**) ZLP FWTM relative error. Corresponding equations are shown on each plot (Eq. S1–S4).

All mentioned parameters are measured for 4000 randomly generated spectra with various OTF, and noise modulations (Table S2). As demonstrated in Fig. 5, EELSpecNet achieves drastically better performance in retrieving zero loss peak shape and removing its tail compared to RL, both visually (Fig. 5a) and quantitatively (Fig. 5b–e). In this regard, while RL Bayesian method restores ZLP tail’s FWHM and FWTM with a recovery rates of around respectively $70\%$ and $82\%$ at best, after 100 RL iterations (Fig. 5b,c), EELSpecNet recovers FWHM and FWTM of the ZLP by more than $99.9\%$ (Fig. 5b,c). The results of each restored signal is also compared with the ZLP of the original signal. As is demonstrated using violin plots in Fig. 5d,e, the EELSpecNet error in reconstructing ZLP is less than $0.6\%$ for both FWHM and FWTM, which is not comparable with the over $100\%$ error observed in RL method. The stability of EELSpecNet at different OTF broadening can also be seen in the performance variation of EELSpecNet which is below $0.5\%$ in reconstructing FWHM and FWTM (magnified orange violin plots, Fig. 5b–e). These result show how EELSpecNet is capable of fully removing the ZLP tail in a NZ-EELS signal. In this regard, as is shown in Fig. S5, the ZLP and its tail can be fully removed from the signal by generating a proper training set.

In order to evaluate the fidelity of the entirety of the reconstructed signal to its original reality, a structural similarity measure (SSIM), introduced by Wang et al.^65,66^, is implemented. As this measure is widely used, evaluated, and validated for images^65,66,66–69^, each spectrum in this work is converted to an energy resolved color map (Fig. S6). According to the SSIM evaluation displayed as violin plots for 2000 randomly generated spectra (Table S3), other than higher signal fidelity in the U-CNN-reconstructed signal, the deep learning solution shows less variance among evaluation set. Similar evaluations are presented using the mean squared error in Fig. S7.

**Figure 6:**
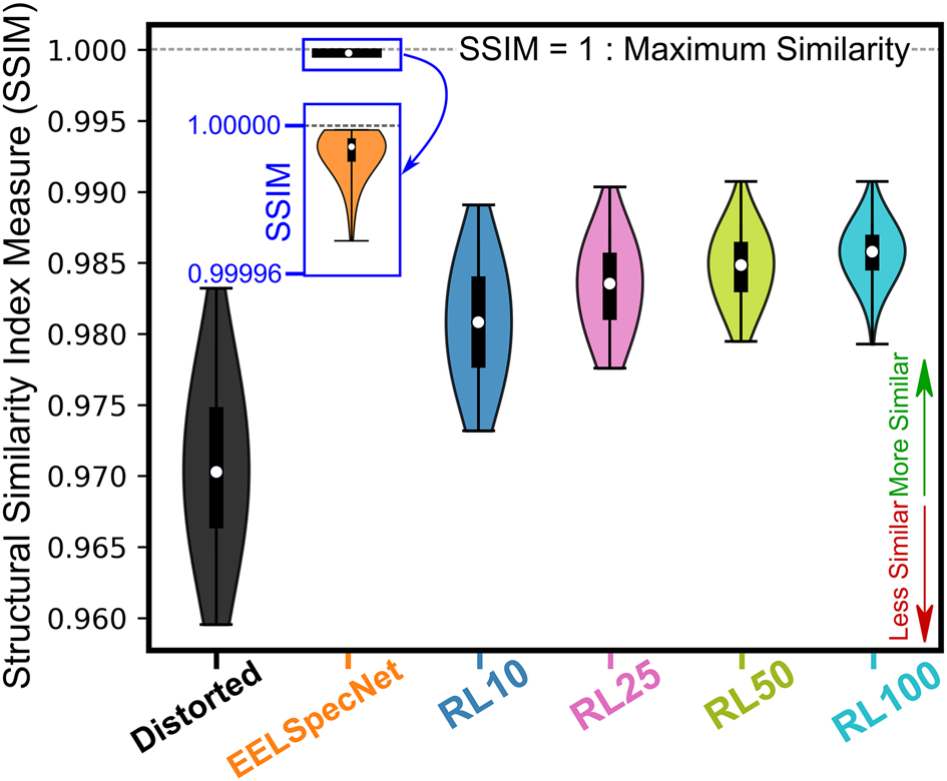
Violin plot representing the structural similarity index measure (SSIM), calculated for distorted and deconvolved spectra. The original spectra are set as the reference point.

**Figure 7:**
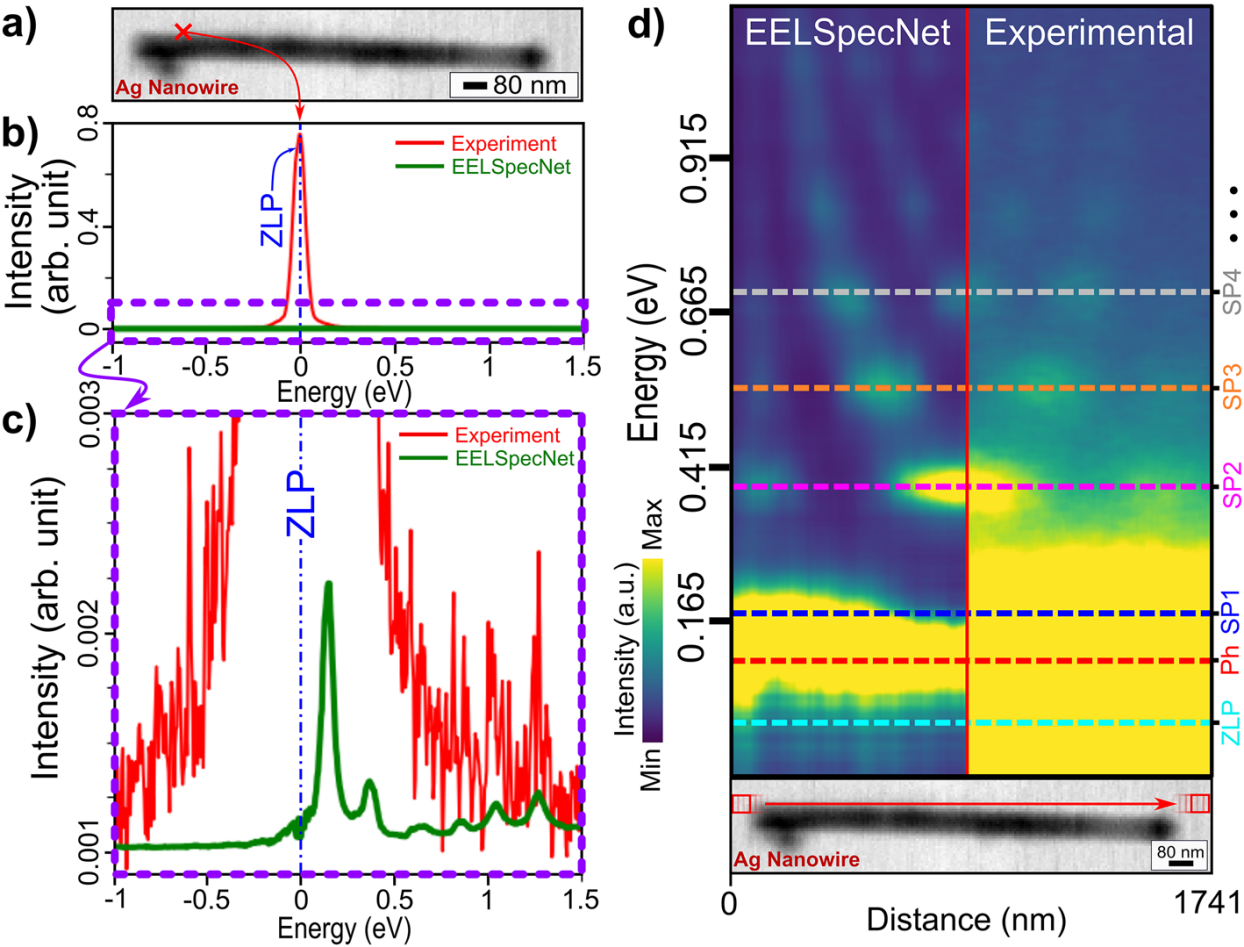
(**a**) Bright-field Scanning transmission electron microscope (BF-STEM) image of a silver nanowire. (**b**) A single NZ-EELS spectra captured from a point near the silver nanowire tip (red curve) and its EELSpecNet reconstruction signal (green curve). (**c**) Magnified experimental and EELSpecNet-restored spectra demonstrated in (**b**). (**d**) EELS map of the silver nanowire shown in (**a**). The map consists of averaged spectra capture along the nanowire (red arrow illustrated on the BF-STEM micrograph). The left side of the map contains EELSpecNet-restored data and the right side demonstrates the experimentally obtained spectra. Surface plasmon modal evolution in energy is marked for a few low energy modes (SP1 to SP4). A phonon peak related to the SiN substrate is also revealed in EELSpecNet-restored map at around 0.1 eV (marked as “Ph” on the map).

Even though a quantitative evaluation of a deconvolution method on an experimentally obtained data may not be possible, it is still reasonable to examine qualitatively the output of a deconvolution process on a real case of study. In this work, an experimentally obtained NZ-EELS data from a silver nanowire is used to investigate EELSpecNet’s performance on the real data (Fig. 7). The result of the EELSpecNet deconvolution on a single spectrum of an obtained hyperspectral NZ-EELS data set is shown in Fig. 7a–c as an example. As is demonstrated in Fig. 7c, the U-CNN network was trained to fully remove the ZLP and its tail (similar to Fig. S5). The restored signal (green curve) obviously shows less high-frequency noise artifacts and, unlike experimental data, the restored signal contains well-defined feature peaks (surface plasmon polariton peaks). In order to find whether suggested peaks by EELSpecNet are physically meaningful, the local distribution of surface plasmon polariton (SPP) hot spots (also known as modal SPP evolution map) is used as a reference^70,71^. Therefore, the whole hyperspectral data demonstrated in Fig. 7a is restored by EELSpecNet. The map of modal evolution of SP resonances in the silver nanowire is obtained by averaging spectra over a 7 pixels by 7 pixels areas along the red arrow in the bright-field scanning transmission electron microscope (BF-STEM) image in Fig. 7d; these EELS maps are obtained for both experimental data and EELSpecNet-restored spectral data. As is depicted in Fig. 7d, the modal distribution of SPs obtained from experimental EELS map fully matches the EELSpecNet-restored data, which confirms that in addition to ZLP tail removal and noise cancellation, the peaks (hot spots) restored by EELSpecNet are qualitatively loyal to their physical origin.

In addition to surface plasmon modes, EELSpecNet-restored map spotted a phenomenon at energies below the the energy of the first surface plasmon mode (Fig. 7d, “SP1”), at around 0.1 eV (Fig. 7d, “Ph”). Due to the fact that a 30 nanometer-thick silicon nitride (SiN) substrate (TEM grid) was used for conducting this experiment, this activity was identified as a phonon excitation in the SiN film. The energy of the restored phonon peak in this work, is fully aligned with the values reported for SiN phonon excitation in the literature^72–74^. Because of the vicinity of the SiN phonon peak to ZLP and the silver nanowire’s SP1 peak (dipole mode), spotting this peak in the raw experimental data would have been practically impossible.

**Figure 8:**
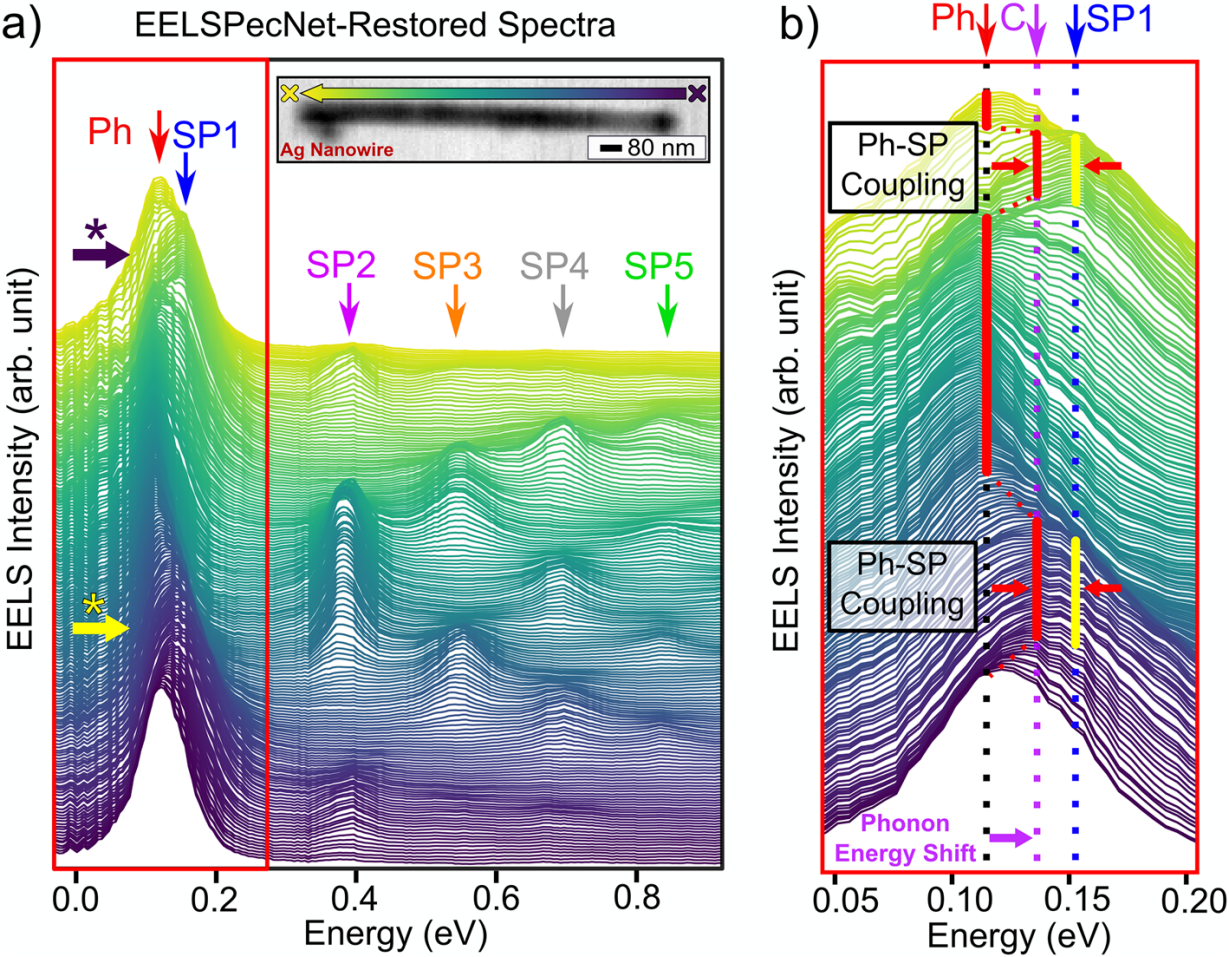
(**a**) EELSpecNet-restored NZ-EELS spectra extracted from along the silver nanowire, as is shown in the BF-STEM image on the top-right. (**b**) Magnified spectra around the 0.1 eV (as is shown in the red box in (**a**)). SiN phonon excitation, first surface plasmon mode in silver, and the shifted (coupled) energy state of the SiN phonon excitation are respectively shown by “Ph”, “SP1”, and “C” signs.

To better demonstrate the behaviour of the SiN phonon peak, EELSpecNet-restored NZ-EELS spectra from along the silver nanowire are depicted in Fig. 8a. It is fascinating that the deep learning solution, beyond revealing the SiN phonon peak, can also detect detailed spectral features and variations within a 40 meV energy window as is magnified in Fig. 8b. The energy shifts of the phonon peak when it is collocated with the SP1 (dipole) surface plasmon mode (near the two tails of the silver nanowire), is highlighted in Fig. 8. As is shown in Fig. 8b, the interaction of the surface plasmon dipole mode (SP1) and the phonon excitation, results in formation of a new coupled energy state in the system (the new energy state is marked as “C” in Fig. 8) consistent with results obtained at much higher energy resolution^75–78^.

Although further discussion about the quality and details of this phonon-plasmon coupling is subject of the future work, it is worth highlighting the advantage of EELSpecNet deep neural network in demonstrating spectral features that were not expected prior to the signal restoration.

Overall, EELSpecNet performance evaluation strongly suggest that deep learning signal reconstruction solutions are extremely efficient and consistent in cleaning hyperspectral signals from high frequency noise, and retrieving information buried under the major components (modulations) of a signal, such as ZLP tail in the case of NZ-EELS, upon sufficient training of the network. In addition, deep convolutional neural networks are, by far, more robust than conventional statistical approaches such as Bayesian RL algorithm in extracting and dealing with complex signal modulations.

## Summary and conclusion

In hyperspectral imaging techniques, such as NZ-EELS, different parameters from instrumental aberrations and imperfections, to nearby electromagnetic interference considerably affect and distort the output data in unpredictable ways. The importance of understanding the reality behind these distorted data drives the efforts shown in the numerous studies conducted for retrieving the origin of this information. The ultimate goal in this regard is to be able to automate microscopy systems to provide outputs that are not affected by the artifacts left from instrumental imperfections or alignments.

Based on the results provided in this study, the full potential of U-CNN deep neural networks can be a prelude to have fast responding reality reconstruction machines that can be implemented on the microscopes or be used for advanced processing of the experimentally captured data. In this regard, a deep U-CNN network named EELSpecNet is introduced and its capability in restoring physical reality of hyperspectral signals (specifically NZ-EELS signals in this work) is evaluated here.

As is demonstrated in Figs. 4 and S4, the deep learning solution is extremely efficient and robust in HF noise cancellation in different conditions; at extremely high noise amplitudes it is shown that EELSpecNet reduces the noise by a factor of 40 (Fig. 4a). EELSpecNet also proved to be extremely efficient in ZLP tail suppression (Fig. 5), and is even capable of fully removing ZLP traces from the signal (Fig. S5). With respect to the fidelity of the restored signal to the original signal, two measures used (structural similarity index measure (SSIM, Fig. 6) and mean squared error (MSE, Fig. S7)) show EELSpecNet’s ability in replicating features in the original signal. Based on the SSIM index measurements, EELSpecNet-restored signals that have more than $99.99\%$ of structural similarity to their original reality (Fig. 6).

Successful implementation of the trained EELSpecNet architecture for deconvolving an experimentally obtained NZ-EELS data proves the effectiveness of EELSpecNet performance, and also the advantage of the generalized-learning strategy used in this work in revealing spectral complexities and even unforeseen phenomena (Figs. 7, and 8). Although the training process and hyper-parameter tuning (number of epochs, size of the training data set, optimizer used, etc.) may change in different condition (based on the users’ needs), this work reveals the impressive capability of the EELSpecNet neural network in learning spectral complexities. The promising results presented in this study may facilitate quantitative analysis of hyperspectral data that suffer from significant artifacts or are restricted by experimental limitations (low dose spectroscopy, ultra fast microscopy, etc.).

Due to the physics-independent approach used in this work, EELSpecNet deconvolving U-CNN could potentially be applied to other spectroscopy techniques. As in every deep learning process, however, the performance is closely related to the quality of the training data that the user provides and the learning process (hyperparameters tuning). Of course, implementation of this U-CNN solution for different areas may require other domain-specific evaluations, beyond what is suggested in this research. Indeed, users must be aware that neural networks are to understand the features and the relationships between them within a training set and shall not be used as a solution to all conditions without proper training. By training the EELSpecNet neural network on larger data sets and adding more complexity to the training set, better transfer learning capabilities are assumed for this U-CNN in the future.

## Methods

### Architecture

The architecture used in this work as is demonstrated in Fig. 1 is a U-shape fully convolutional neural network (U-CNN) with skip connections. This convolutional autoencoder-like architecture is inspired by the U-net architecture used for segmentation of bio-images^39,42,43^. The main network used for evaluation in this study has 10 decoding and 10 encoding layers, however, the number of layers can change based on the users need and size of the input signals. In this regard, the U-CNN network used for experimental data reconstruction (Figs. 7 and 8) had 9 encoding and decoding layers, as the experimental signal did not have enough channels to use the whole depth of the network. The layers in the decoding path are convolutional neural network layers and the encoding path consists of convolution-transverse network. Other than the last layer which uses a positive tanh activation function (Fig. S8), all layers use rectified linear unit (ReLu) activation function. The skip connection is, in fact, concatenations of different output tensors between decoding and encoding path (Fig. 1) to enhance the learnability of the network^40,50,79,80^. All the spectra were normalized before being provided to the neural network.

### Data generation

The parameters determining different components of the signals, including number peaks (feature peaks), FWHM of these peaks, etc., are generated randomly, independently, and equally likely within ranges specified in Tables S1, S2, and S3. These generated parameters are used for preparing training and testing data sets. The uniformity of the distribution of the generated parameters in this study, is examined for each data set, and the results are demonstrated in Fig. S9 (for Table S1), Fig. S10 (for Table S2), and Fig. S11 (for Table S3).

### Computational hardware and software

In order to improve accessibility and reproducibility, all codes are scripted in Python and run using open access Google Colaboratory (Colab) GPU and TPU platform. The deep learning portion of the project was performed using Tensorflow2 and Keras in Python^81^. GPU and TPU version of EELSpecNet script in addition to a full list of all dependencies can be found in reference^49^, and on the github repository devoted to this project (https://github.com/shmouses/EELSpecNet).

### Method credibility and failure criteria

As with all deep learning methods, the U-CNN solution used in this study has high bias and low variation with respect to the training data set. In other words, the credibility of the results obtained using this deep learning solution (similar to other deep learning methods) is strongly dependent on the quality, representability, and boundaries of the training data set and training process (hypermeter selection). Therefore, in the case of this study, the reconstruction fails (leads to inaccuracies) when the provided spectra sit beyond the scope of the training set. For instance, if a deep neural network is trained to deconvolve Lorentzian-type peaks, using it for a signal containing gaussian-type peaks will lead to some inaccuracies. As another example, having artifacts more or less intense than what is predicted in the training process can also lead to imprecision and failure of the model.

Of course, deep learning solution has some intrinsic artifacts such as pixel-limited information interpretability due to the discretization process. In addition, based on findings in this study (Fig. 4) there is always a residual noise in the reconstructed spectra that is scaling with the intensity of the noise in the distorted signal. Despite the great performance of EELSpecNet, there is still a signal-to-noise ratio detection limit that corresponds to the black curve in Fig. 4. Similar observations are also noticeable in the results presented in Figs. 5, 6, and S7.

In a nutshell, it is advised to the users to always train the network according to their application. The current study focuses on typical plasmonic and phononic activities in the proximity of the zero-loss peak.

### Sample and microscope parameters

The experimental results are obtained using a monochromated Thermo Fisher Scientific Titan 80-300 scanning transmission electron microscope (STEM) operating at a voltage of 80 keV. Near zero-loss electron energy loss spectroscopy (NZ-EELS) is conducted in Ultimono^TM^ mode and the optimum energy resolution in vacuum for the experimentally captured data is 45 meV. The silver nanowires suspended in isopropyl alcohol is purchased from Sigma-Aldrich and is dropcast on a SiN TEM grid.

### Data availability

The Python programs for generating datasets used in this study are available in the EELSpecNet’s GitHub repository at https://github.com/shmouses/EELSpecNet. The files containing the generated and experimental data, due to the large size of the files and memory limitations of data-sharing platforms, are available from the corresponding authors upon reasonable request.

## Supplementary Information

Supplementary Information.
