# Supplementary material for: Alignment-invariant signal reality reconstruction in hyperspectral imaging using a deep convolutional neural network architecture
Source: Sci Rep. 2022 Oct 19;12:17462. doi: 10.1038/s41598-022-22264-3 (PMC9581942; doi:10.1038/s41598-022-22264-3)
Supplement: Supplementary file 1 — Supplementary Information. [file 41598_2022_22264_MOESM1_ESM.pdf]

Alignment-Invariant Signal Reality Reconstruction in Hyperspectral Imaging Using a Deep Convolutional Neural Network Architecture

S. Shayan Mousavi M.[1], Alexandre Pofelski[2], Hassan Teimoori[3], and Gianluigi A. Botton[1, 4]

[1] McMaster University, Materials Science and Engineering, Hamilton, L8S 4L8, Canada
[2] Brookhaven National Laboratory, Upton, NY 11973, USA
[3] McMaster University, Walter G. Booth School of Engineering Practice and Technology, Hamilton, L8S 4M1, Canada
[4] Canadian Light Source, Saskatoon, S7N 2V3, Canada

Supplementary Information

This document provides supplementary information to the article published with a similar title. The document is organized in a manner that first presents supplementary figures, then tables, and, finally, equations.

Supplementary Figures:

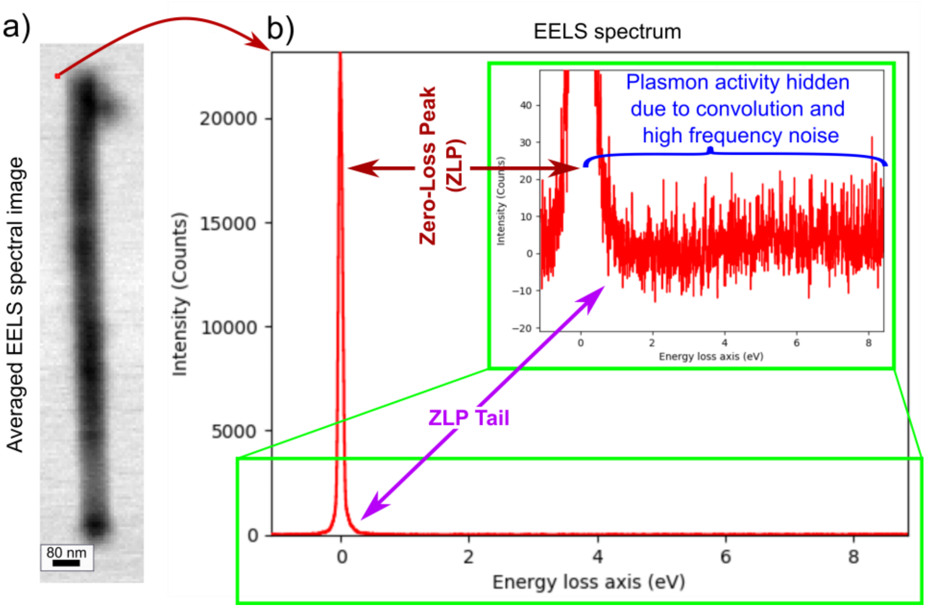

Figure S1. a) A bright-field STEM image formed by EELS spectra mapped around a silver nanowire. b) An example of a spectrum extracted from a pixel near the tip of the nanowire (red box and arrow). Low energy features, such as plasmonic activities shown in the magnified region (green box), are distorted and buried under the high-frequency noise.

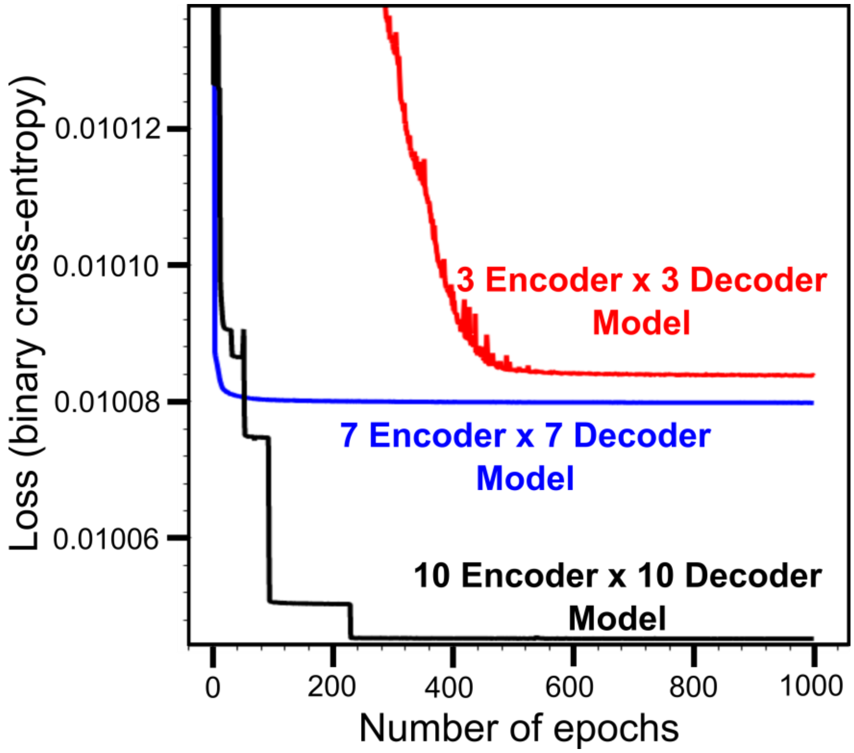

Figure S2. Neural network training loss of EELSpecNet U-CNN of different depths at each epoch. As is demonstrated, the deeper network with 20 layers (10-by-10 network) shows lower losses (better convergence) per epoch.

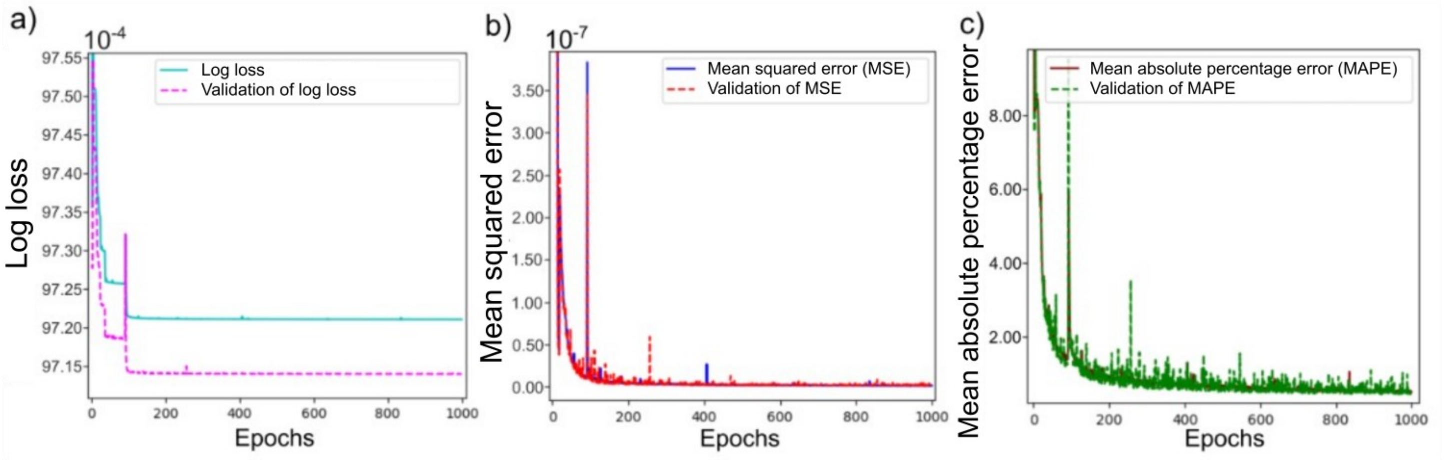

Figure S3. a) log loss measured at each epoch during EELSpecNet 10-by-10 U-CNN learning and its validation. b), and c) respectively demonstrated the mean square error (MSE) and the mean absolute percentage error (MAPE) measured at each epoch during learning as a secondary measure to monitor the progress in the 10-by-10 network training.

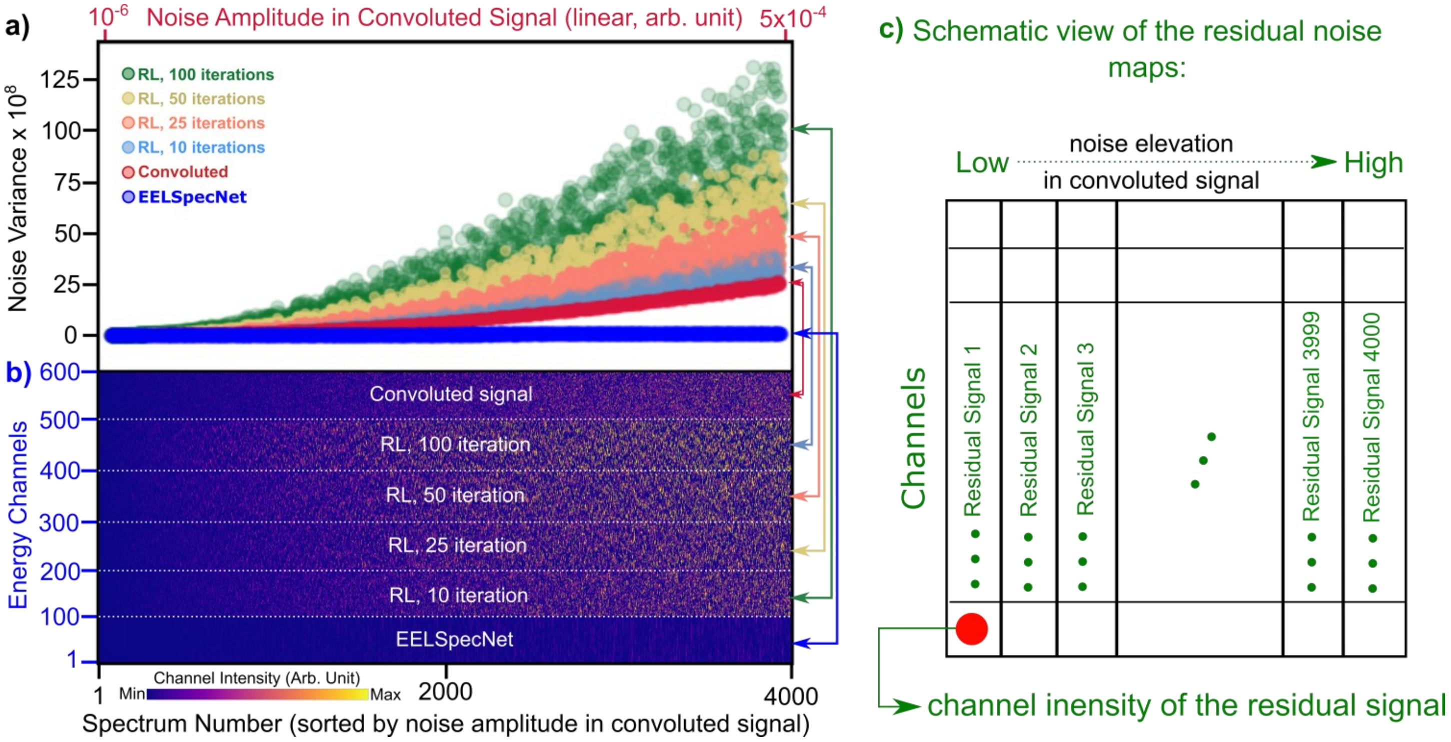

Figure S4. a) Demonstrates the noise variance evolution in distorted (convoluted) and restored signals while developing higher noise amplitudes in the distorted (convoluted) signals. b) 100-channel long slices of the residual noise maps generated for 4000 spectra used for studying HF noise-cancelling properties. Each 100 consecutive energy channels shown in (b) (vertical axes) demonstrate a residual signal after applying the mentioned deconvolution technique. These spectra are sorted based on the noise amplitude in the distorted (convoluted) data from left to right. c) The schematic view of how residual noise maps are created in this work.

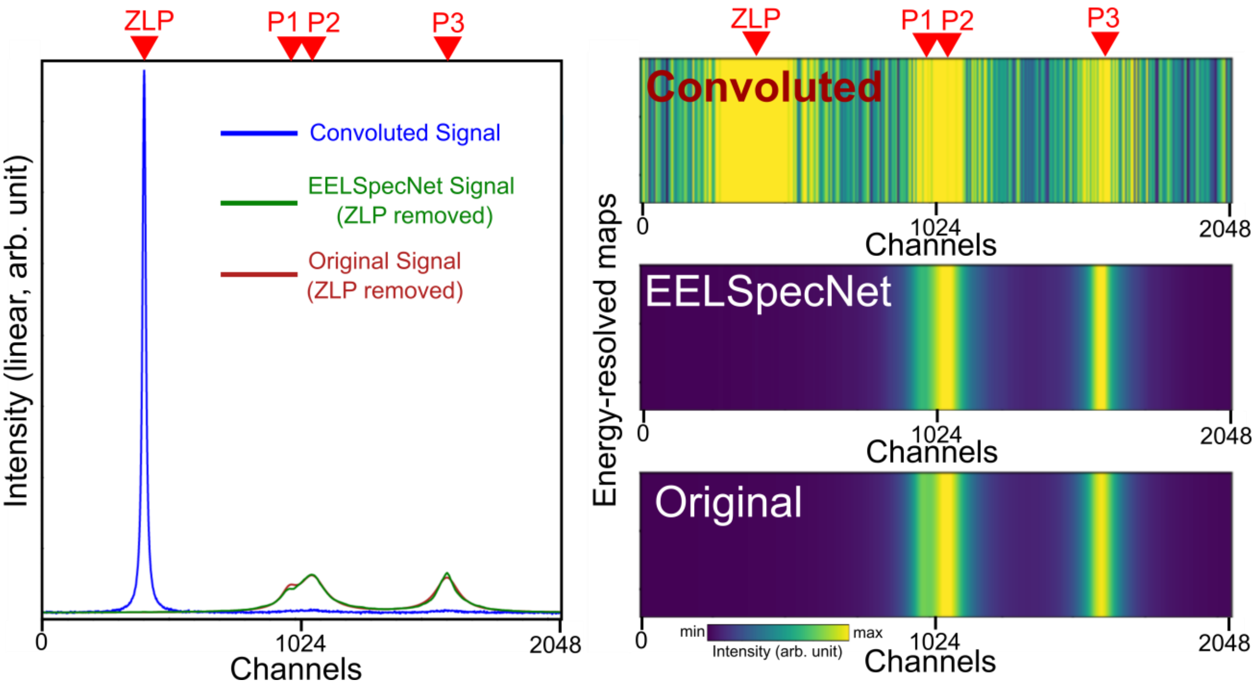

Figure S5. An example EELSpecNet U-CNN trained for deconvolving (restoring) NZ-EELS signal while fully removing ZLP-related effects (ZLP, ZLP tail, and broadening effects). Distorted (convoluted), restored, and original spectra are demonstrated on the left and corresponding energy-resolved maps are shown on the right.

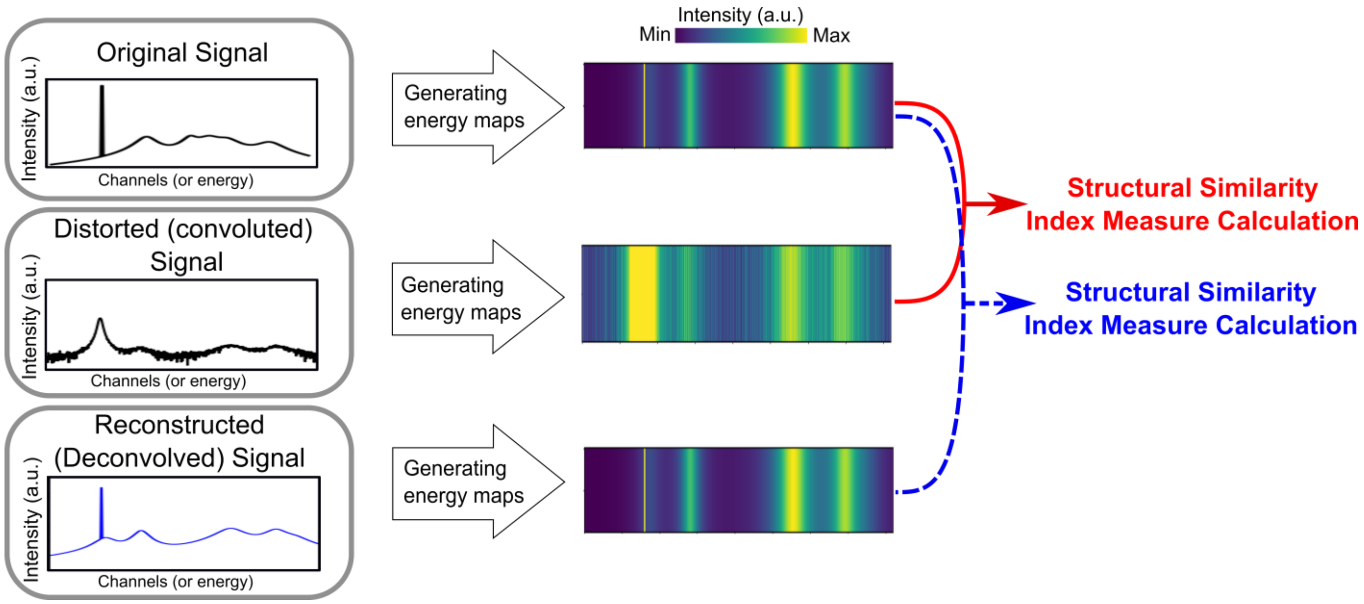

Figure S6. A schematic view of the data processing procedure used for calculating structural similarity index measure calculation (SSIM) on different signal sets.

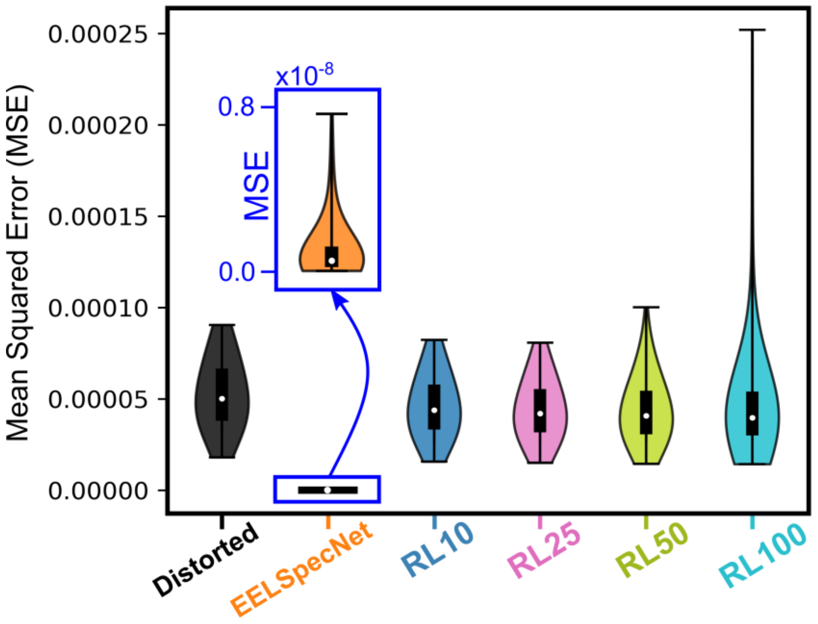

Figure S7. Violin plots demonstrate the mean squared error distance of the distorted and restored signals from the original signals.

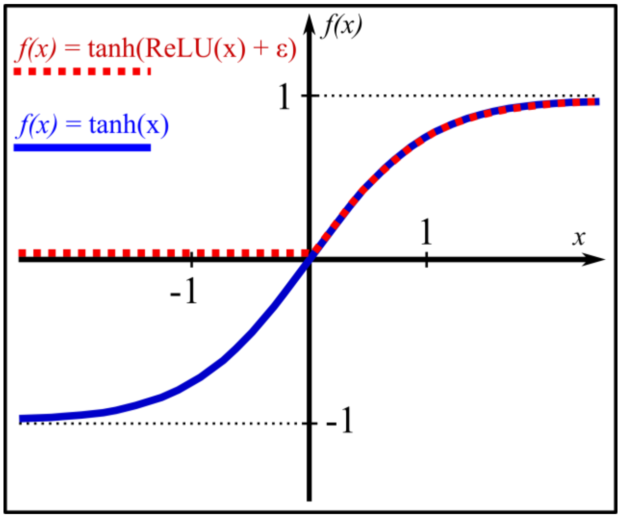

Figure S8. The behaviour of the positive tanh activation function used in this study is demonstrated using the red dotted line. The value ε is an infinitesimal value to guarantee that the function output is always positive.

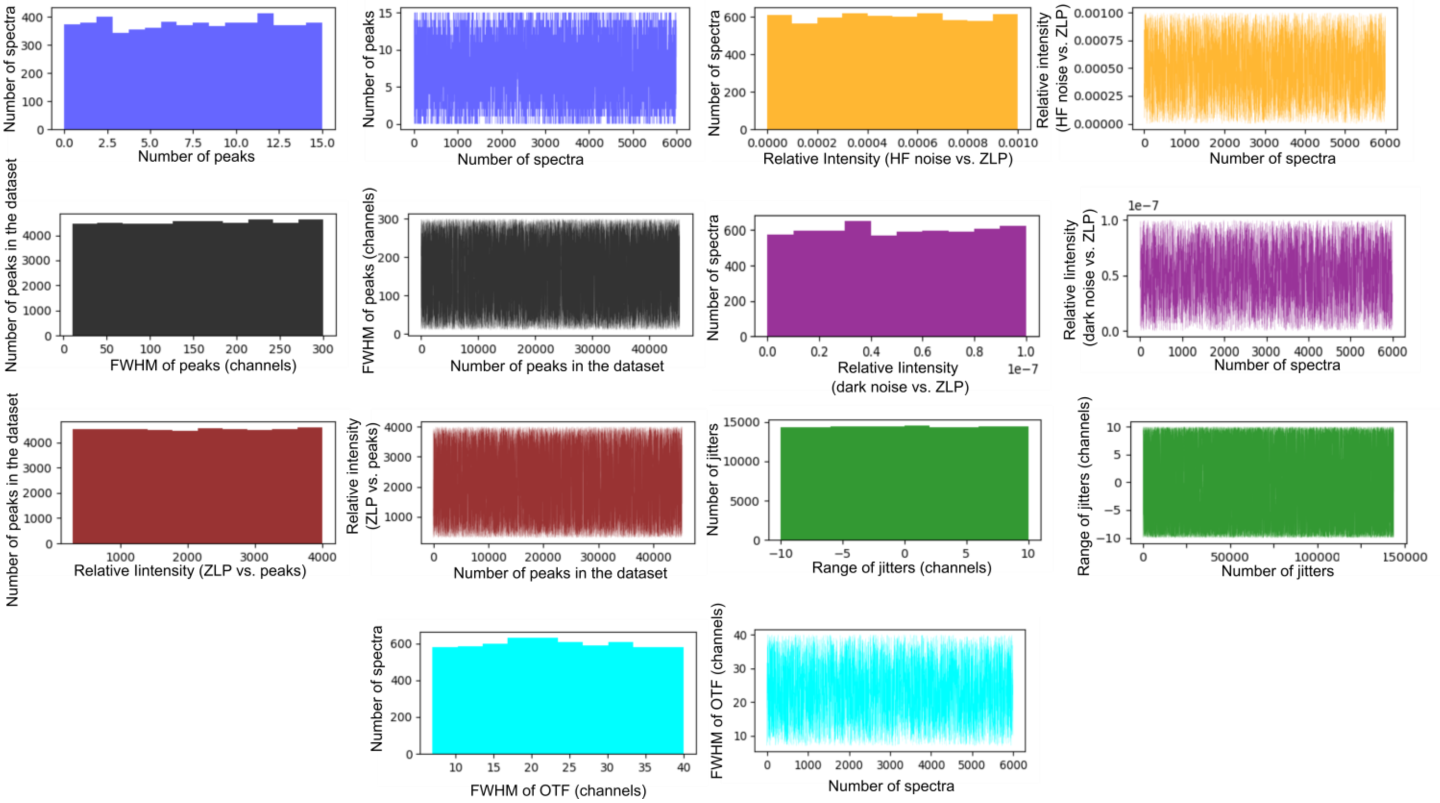

Figure S9. The statistical distribution of randomly generated parameters for 6000 signals (spectra) in the training dataset (Table S1). Each distribution is presented in a pair (similar colours) to evaluate the uniform distribution of the generated data. In each pair, the left plot is the histogram of the data distribution on the right.

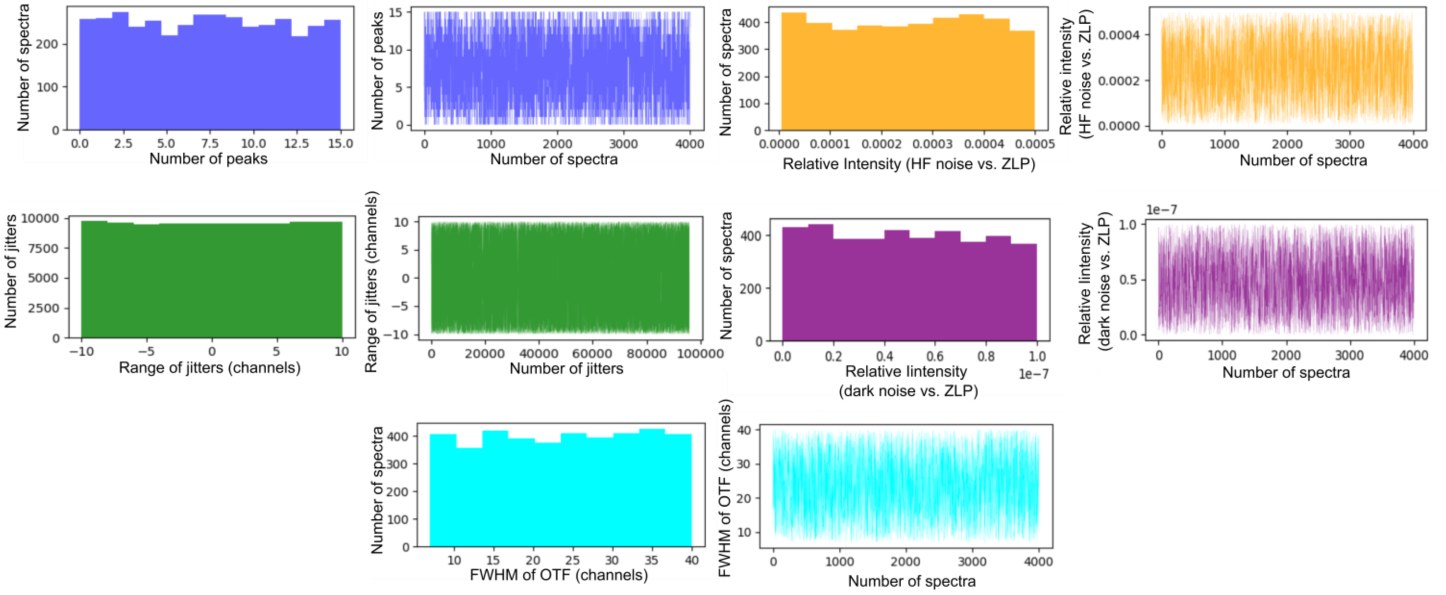

Figure S10. The statistical distribution of randomly generated parameters for 4000 signals (spectra) in the evaluation dataset (Table S2). Each distribution is presented in a pair (similar colours) to evaluate the uniform distribution of the generated data. In each pair, the left plot is the histogram of the data distribution on the right.

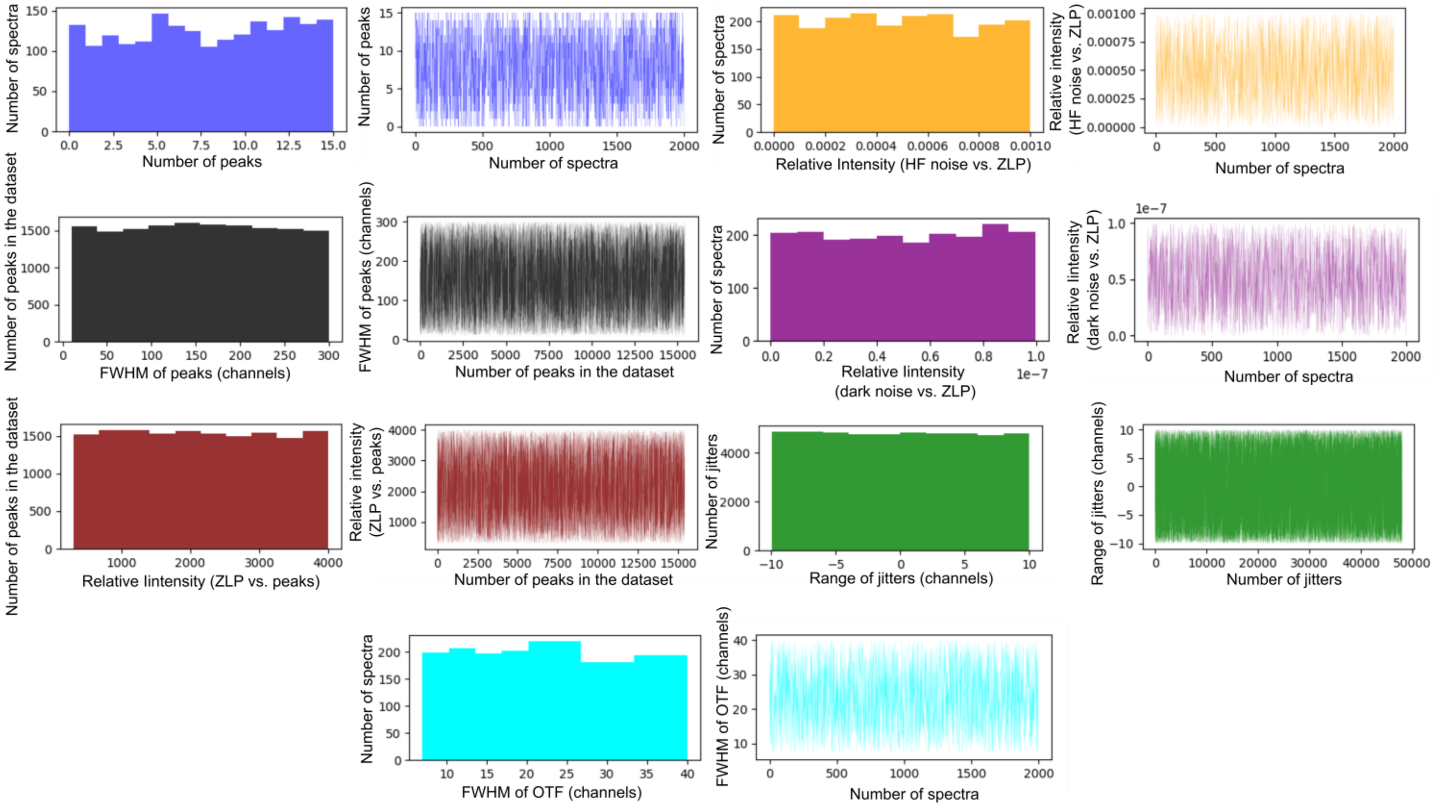

Figure S11. The statistical distribution of randomly generated parameters for 2000 signals (spectra) in the evaluation dataset (Table S3). Each distribution is presented in a pair (similar colours) to evaluate the uniform distribution of the generated data. In each pair, the left plot is the histogram of the data distribution on the right.

Supplementary Tables:

Table S1: The ranges of the parameters used for generating 6000 NZ-EELS spectra for general training and training validation of EELSpecNet

Number of feature peaks	FWHM of feature peaks (channels)	Relative intensity of ZLP vs. feature peaks	Relative HF noise intensity (vs. ZLP intensity)	Relative dark noise intensity (vs. ZLP intensity)	System instability (beam jitters)	Range of channel jitters of the beam vs. original coordinate (channels)	Optical transfer function (OTF) FWHM broadening (channels)	Number of Spectra
< 16	10 – 300	300-4000	10^{-8} – 10^{-3}	0 – 10^{-7}	25 random energy jitters (jumps) per spectrum	±10	7 – 40	6000

Table S2: The ranges of the parameters used for generating 4000 featureless NZ-EELS spectra to evaluate noise cancelling, and ZLP tail removal quality in the restored signal

Number of feature peaks	FWHM of feature peaks (channels)	Relative intensity of ZLP vs. feature peaks	Relative HF noise intensity (vs. ZLP intensity)	Relative dark noise intensity (vs. ZLP intensity)	System instability (beam jitters)	Range of channel jitters of the beam vs. original coordinate (channels)	Optical transfer function (OTF) FWHM broadening (channels)	Number of Spectra
0	N/A	N/A	5×10^{-6} – 5×10^{-4}	0 – 10^{-7}	25 random energy jitters (jumps) per spectrum	±10	7 – 40	6000

Table S3: The ranges of the parameters used for generating 2000 NZ-EELS spectra for evaluating the fidelity (similarity) of the restored signals to the original signal

Number of feature peaks	FWHM of feature peaks (channels)	Relative intensity of ZLP vs. feature peaks	Relative HF noise intensity (vs. ZLP intensity)	Relative dark noise intensity (vs. ZLP intensity)	System instability (beam jitters)	Range of channel jitters of the beam vs. original coordinate (channels)	Optical transfer function (OTF) FWHM broadening (channels)	Number of Spectra
< 16	10 – 300	300-4000	10^{-8} – 10^{-3}	0 – 10^{-7}	25 random energy jitters (jumps) per spectrum	±10	7 – 40	2000

Supplementary Equations:

$$FWHM\ Recovery\ Rate = \frac{|\mathrm{FWHM}_{\mathrm{Dst.}} - \mathrm{FWHM}_{\mathrm{Rst.}}|}{\mathrm{FWHM}_{\mathrm{Dst.}} - \mathrm{FWHM}_{\mathrm{Org.}}} \times 100 \qquad \text{(Eq. 1)}$$

$$FWTM\ Recovery\ Rate = \frac{|\mathrm{FWTM}_{\mathrm{Dst.}} - \mathrm{FWTM}_{\mathrm{Rst.}}|}{\mathrm{FWTM}_{\mathrm{Dst.}} - \mathrm{FWTM}_{\mathrm{Org.}}} \times 100 \qquad \text{(Eq. 2)}$$

$$FWHM\ Relative\ Error = \frac{|\mathrm{FWHM}_{\mathrm{Rst.}} - \mathrm{FWHM}_{\mathrm{Org.}}|}{\mathrm{FWHM}_{\mathrm{org.}}} \times 100 \qquad \text{(Eq. 3)}$$

$$FWTM\ Relative\ Error = \frac{|\mathrm{FWTM}_{\mathrm{Rst.}} - \mathrm{FWTM}_{\mathrm{Org.}}|}{\mathrm{FWTM}_{\mathrm{org.}}} \times 100 \qquad \text{(Eq. 4)}$$

$\mathrm{FWHM}_{\mathrm{Dst.}}$: Full width at half maximum of the distorted spectrum's ZLP

$\mathrm{FWHM}_{\mathrm{RSt.}}$: Full width at half maximum of the restored spectrum's ZLP

$\mathrm{FWHM}_{\mathrm{Org.}}$: Full width at half maximum of the original spectrum's ZLP

$\mathrm{FWTM}_{\mathrm{Dst.}}$: Full width at tenth maximum of the distorted spectrum's ZLP

$\mathrm{FWTM}_{\mathrm{RSt.}}$: Full width at tenth maximum of the restored spectrum's ZLP

$\mathrm{FWTM}_{\mathrm{Org.}}$: Full width at tenth maximum of the original spectrum's ZLP
